# Comparison of Circulating Markers and Mucosal Immune Parameters from Skin and Distal Intestine of Atlantic Salmon in Two Models of Acute Stress

**DOI:** 10.3390/ijms22031028

**Published:** 2021-01-21

**Authors:** Brankica Djordjevic, Byron Morales-Lange, Charles McLean Press, Jake Olson, Leidy Lagos, Luis Mercado, Margareth Øverland

**Affiliations:** 1 Department of Animal and Aquaculture Sciences, Faculty of Biosciences, Norwegian University of Life Sciences, 1430 Ås, Norway; leidy.lagos@nmbu.no (L.L.); margareth.overland@nmbu.no (M.Ø.); 2 Department of Preclinical Sciences and Pathology, Faculty of Veterinary Medicine, Norwegian University of Life Sciences, 1430 Ås, Norway; charles.press@nmbu.no; 3 Department of Animal and Dairy Sciences, University of Wisconsin, Madison, WI 53706, USA; jmolson22@wisc.edu; 4 Grupo de Marcadores Inmunológicos en Organismos Acuáticos, Pontificia Universidad Católica de Valparaíso, 2950 Valparaíso, Chile; luis.mercado@pucv.cl

**Keywords:** *Salmo salar*, hypoxia, crowding stress, plasma, skin mucus, distal intestine, phenotypic parameters

## Abstract

Ensuring salmon health and welfare is crucial to maximize production in recirculation aquaculture systems. Healthy and robust mucosal surfaces of the skin and intestine are essential to achieve this goal because they are the first immunological defenses and are constantly exposed to multistressor conditions, such as infectious diseases, suboptimal nutrition, and environmental and handling stress. In this work, Atlantic salmon, split from a single cohort, were subjected to acute hypoxia stress or 15-min crowding stress and observed over a 24-h recovery period. Samples were collected from fish at 0, 1, 3, 6, 12 and 24 h post-stress to analyze plasma-circulating markers of endocrine function (cortisol), oxidative stress (glutathione peroxidase) and immune function (interleukin 10 (IL-10), annexin A1). In addition, mucosal barrier function parameters were measured in the skin mucus (Muc-like protein and lysozyme) and distal intestine (simple folds, goblet cell size and goblet cell area). The results showed that both acute stress models induced increases of circulating cortisol in plasma (1 h post-stress), which then returned to baseline values (initial control) at 24 h post-stress. Moreover, the hypoxia stress was mostly related to increased oxidative stress and IL-10 production, whereas the crowding stress was associated with a higher production of Muc-like protein and lysozyme in the skin mucus. Interestingly, in the distal intestine, smaller goblet cells were detected immediately and one hour after post-hypoxia stress, which could be related to rapid release of the cellular content to protect this organ. Finally, the correlation of different markers in the hypoxic stress model showed that the circulating levels of cortisol and IL-10 were directly proportional, while the availability of Muc-like proteins was inversely proportional to the size of the goblet cells. On the other hand, in the crowding stress model, a proportional relationship was established between plasma cortisol levels and skin mucus lysozyme. Our results suggest key differences in energy partitioning between the two acute stress models and support the need for further investigation into the interplay of multistressor conditions and strategies to modulate immunological aspects of mucosal surfaces.

## 1. Introduction

Continuous growth of the aquaculture industry and implementation of recirculating aquaculture systems (RAS), where fish are kept in a closed environment during the whole production cycle, raise concerns with regard to fish welfare, health and growth potential [1,2]. Fish management practices can involve multiple stressful conditions (chemical, biological, physical and procedural) that can affect the responses of mucosal surfaces as the first defense barriers against biological challenges [1,2,3,4]. The ability of fish to cope with stress depends on species, stage of development, age, and the degree of habituation to the stressors [5]. However, stress response assessment is a complex topic and measuring and interpreting the results is not always clear for a variety of reasons, including differences in stress model procedures among experiments [6]. There are numerous studies that have looked at different stress models and evaluated stress responses at diverse levels [7,8,9,10,11]. However, few studies have performed side-by-side characterizations of acute stress effects on mucosal tissues such as skin and intestine. 

Many studies have suggested negative effects resulting from higher stocking densities on fish growth and welfare, mostly focusing on physiological stress responses [12,13]. Additionally, the negative effects of fish being exposed to acute hypoxia, which often occurs in commercial aquaculture units, have been recognized and documented [14]. Exposure to either of these two stressors, or to both simultaneously, may induce morphological and structural changes of different body tissues in fish, which could impact the regulation of digestion, absorption, and overall metabolism [15]. Atlantic salmon and rainbow trout exposed to an acute handling stress showed instant alterations (increased junctional gap) of mid-intestine junctional complexes that were of a transient character and that returned to their normal forms within 24–48 h post-stress [9,16]. However, chronic stress exposure to higher stocking densities [17] demonstrated a decreased physical intestinal barrier, denoted by decreased transepithelial electrical resistance and elevated paracellular permeability, and affected the intestinal immune system, decreasing mRNA expression of interferon gamma (IFNγ)and increasing infiltrations of neutrophils. The effect of acute exposure to higher stocking densities or to acute hypoxia may compromise skin and intestine mucosal responses that have not been entirely explained and that may vary between stressors. 

Therefore, in this study, Atlantic salmon were exposed to one-minute hypoxia or to crowding stress (75 kg m^3^) for 15 min. The overall objective was to document the differences in physiological responses of plasma-circulating markers related to endocrine function (cortisol), oxidative stress (glutathione peroxidase (GPx)) and immunity (interleukin 10 (IL-10); annexin A1 (Anxa1)). In addition, mucosal barrier function parameters were measured in skin mucus (Muc-like protein and lysozyme) and histological assessment of distal intestine (DI) mucosa was performed to evaluate effects of acute stressors on local intestinal tissue responses by morphometric measurements of the length of simple folds, as well as analyzing the area and average size of goblet cells in the simple fold epithelium. 

## 2. Results

### 2.1. Plasma Cortisol

**Figure 1:**
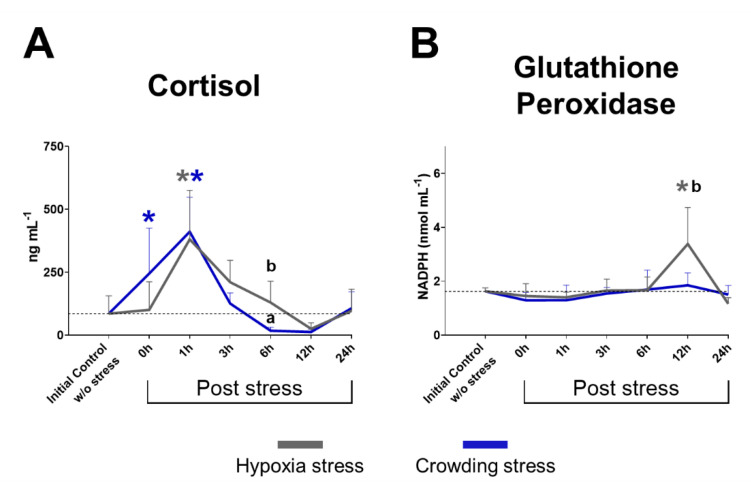
Plasma parameters in samples from Atlantic salmon. (**A**) Cortisol level (ng mL^−1^). (**B**) Glutathione peroxidase (GPx) activity (NADPH (nmol mL^−1^)). Sampling groups: initial control group (without stress) and 0, 1, 3, 6, 12, and 24 h post-stress. In grey, hypoxia stress; in blue, crowding stress. Values are presented as average cortisol values ± standard deviation. Significant differences (*p* < 0.05) are denoted with * (grey *—hypoxia stress group compared with initial control group; blue *—crowding stress group compared with initial control group) and with the letters a and b (compared between stress models).

The results concerning plasma cortisol (Figure 1A) from the initial control fish group (without stress) showed an average of 85.40 ± 71.06 ng mL^−1^, while fish subjected to hypoxia and crowding stress demonstrated changing cortisol levels compared with the initial control. 

The hypoxia stress group showed a significant increase in cortisol level at 1 h post-stress (380.94 ± 194.72 ng mL^−1^, *p*-value = 0.0002). The cortisol level subsequently decreased to a minimum of 25.09 ± 24.0 ng mL^−1^ at 12 h post-stress and at 24 h post-stress returned to values (96.62 ng mL^−1^ ± 86.19, *p*-value = 0.9997) similar to the initial control value. Atlantic salmon subjected to crowding stress demonstrated a high cortisol value immediately after the stressful condition (244.83 ± 180.69 ng mL^−1^, *p*-value = 0.0302 at 0 h). This significantly increased level of cortisol continued to increase, reaching its highest value at 1 h post-stress (410.98 ± 137.52 ng mL^−1^, *p*-value = < 0.0001). The levels of cortisol subsequently declined significantly compared with the hypoxia group at 6 h post-stress (17.63 ± 13.72 ng mL^−1^, *p*-value = 0.0093). The decline in cortisol levels continued until 12 h post-stress (12.67 ± 10.49 ng mL^−1^) before returning to levels similar to the initial control at 24 h post-stress (107.22 ± 65.47 ng mL^−1^, *p*-value = 0.9971).

### 2.2. Glutathione Peroxidase Activity

The glutathione peroxidase (GPx) activity in plasma samples (Figure 1B) showed a significant increase in enzymatic activity at 12 h post-hypoxia stress (3.38 ± 1.35 nmol mL^−1^ of NADPH) compared with both the initial control (1.63 ± 0.13 nmol mL^−1^, *p*-value = < 0.0001) and crowding stress group (1.85 ± 0.45 nmol mL^−1^, *p*-value = 0.0252). GPx activity did not show significant changes at any other time points.

### 2.3. Immunological Markers

**Figure 2:**
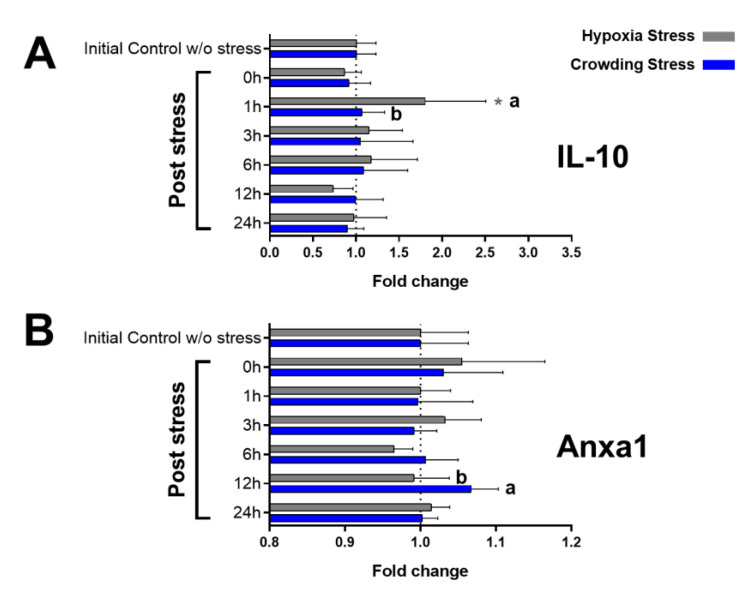
Plasma immunological markers by indirect ELISA (as fold of change relative to the initial control ± standard deviation). (**A**) IL-10. (**B**) Anxa1. Sampling groups: initial control group (without stress) and 0, 1, 3, 6, 12, and 24 h post-stress. In grey, hypoxia stress; in blue, crowding stress. Significant differences (*p* < 0.05) are denoted with * (compared with initial control group) and with the letters a and b (compared between stress models).

The detection of immunological markers obtained by indirect ELISA and expressed as the fold change relative to the initial control showed a significant increase of plasma IL-10 (1.80 ± 0.70) at 1 h post-stress in the hypoxia group (compared with both the initial control, *p*-value = 0.0123, and crowding stress, *p*-value = 0.0372) (Figure 2A). Moreover, Annexin A1 (Anxa1) (Figure 2B) at 12 h post-crowding stress reported an increased level (1.07 ± 0.04) compared with the hypoxia group (*p*-value = 0.0100).

**Figure 3:**
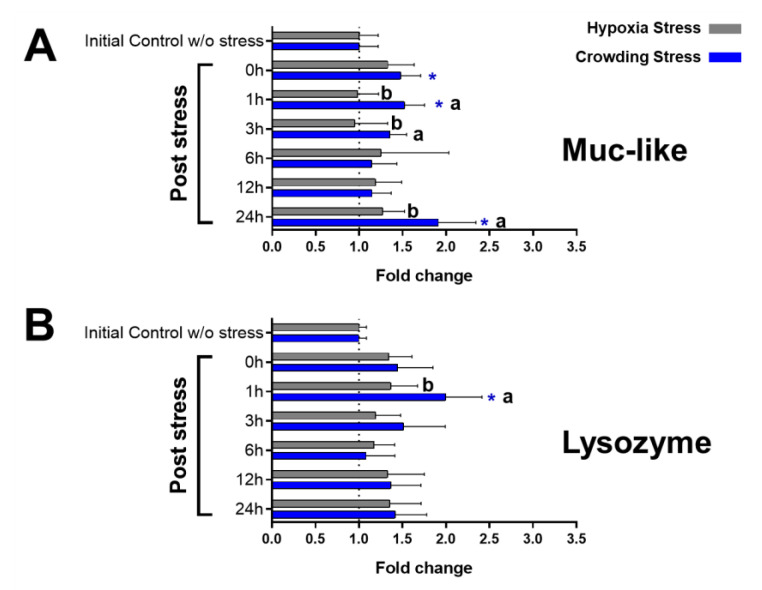
Immunological markers in skin mucus by indirect ELISA (as fold of change relative to the initial control ± standard deviation). (**A**) Muc-like protein. (**B**) Lysozyme. Sampling groups: initial control group (without stress) and 0, 1, 3, 6, 12, and 24 h post-stress. In grey, hypoxia stress; in blue, crowding stress. Significant differences (*p* < 0.05) are denoted with * (compared with initial control group) and with the letters (a and b (compared between stress models).

In skin mucus samples, Muc-like protein (Figure 3A) showed a significantly increased level in the crowding stress group, compared with the initial control, at 0 (1.48 ± 0.23, *p*-value = 0.0202), 1 (1.52 ± 0.23, *p*-value = 0.0097), and 24 h post-stress (1.91 ± 0.43, *p*-value = < 0.0001) and at 1, 3 (1.35 ± 0.19), and 24 h post-stress compared with the hypoxia group (*p*-value = 0.0026, 0.0406, and 0.0106, respectively). Lysozyme levels (Figure 3B) were significantly higher at 1 h post-crowding stress (2.00 ± 0.42) compared with the initial control (*p*-value = 0.0002) and the hypoxia stress groups (*p*-value = 0.0140).

### 2.4. Histology and Morphometry

**Figure 4:**
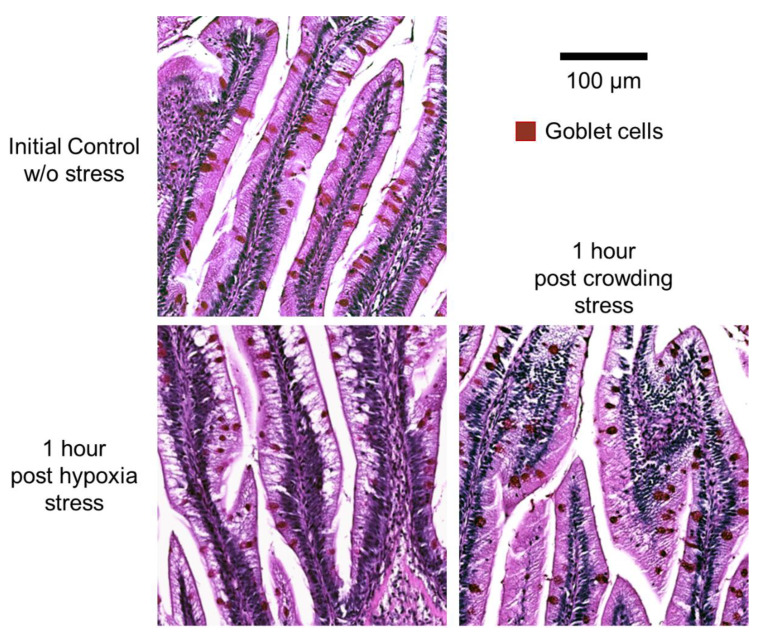
Histology of Periodic acid–Schiff (PAS)-stained tissue sections of distal intestine from Atlantic salmon. Top left panel: initial control group. Lower left panel: 1 h post-hypoxia stress. Lower right panel: 1 h post-crowding stress. In red-brown: PAS+ goblet cells. Bar: 100 µm.

Histological examination of the DI showed a normal structure for the intestinal mucosa in the control, hypoxia, and crowding stress groups. A simple columnar epithelium covered a lamina propria containing blood vessels. The epithelium consisted of vacuolated absorptive epithelial cells and many Periodic acid–Schiff (PAS)-positive (PAS+) goblet cells (Figure 4). An intraepithelial leukocyte population was present. The lamina propria in the simple folds was thin but was broader in the complex folds with the presence of smooth muscle cells.

**Figure 5:**
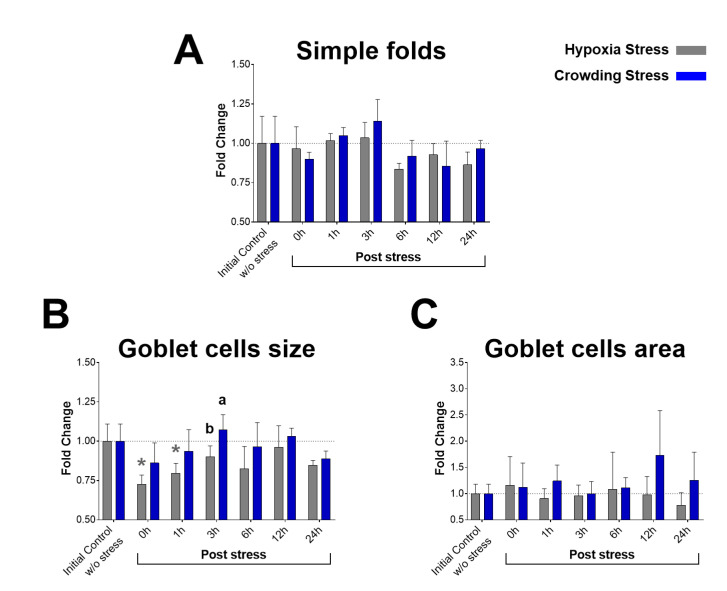
Morphometry of PAS-stained tissue sections of distal intestine from Atlantic salmon. (**A**) Simple folds length ± standard deviation in distal intestine. (**B**) Average size of goblet cells ± standard deviation in simple folds of distal intestine. (**C**) Percentage of area occupied by goblet cells ± standard deviation in epithelium of simple folds. Sampling groups: initial control group (without stress) and 0, 1, 3, 6, 12, and 24 h post-stress. In grey, hypoxia stress; in blue, crowding stress. Significant differences (*p* < 0.05) are denoted with * (compared with initial control group) and with the letters a and b (compared between stress models).

Morphometric examination of simple fold length did not reveal significant differences between the fish groups (Figure 5A). In PAS-stained sections, the area of PAS+ material in the epithelium of simple folds was equated with the presence of goblet cells. The average size of goblet cells (PAS+ particles) was significantly decreased at 0 h (0.73 ± 0.06) and at 1 h (0.80 ± 0.06) post-hypoxia stress compared with the average goblet cell size of the initial control group (1.00 ± 0.11, *p*-value = 0.0029 and 0.0312, respectively) (Figure 5B). In addition, at 3 h post-stress, the average goblet cell size (0.90 ± 0.07) was significantly smaller in the hypoxia group than in the crowding stress group (*p*-value = 0.0285). The percentages of simple fold epithelial area occupied by goblet cells did not show significant differences between the groups.

### 2.5. Correlation

**Figure 6:**
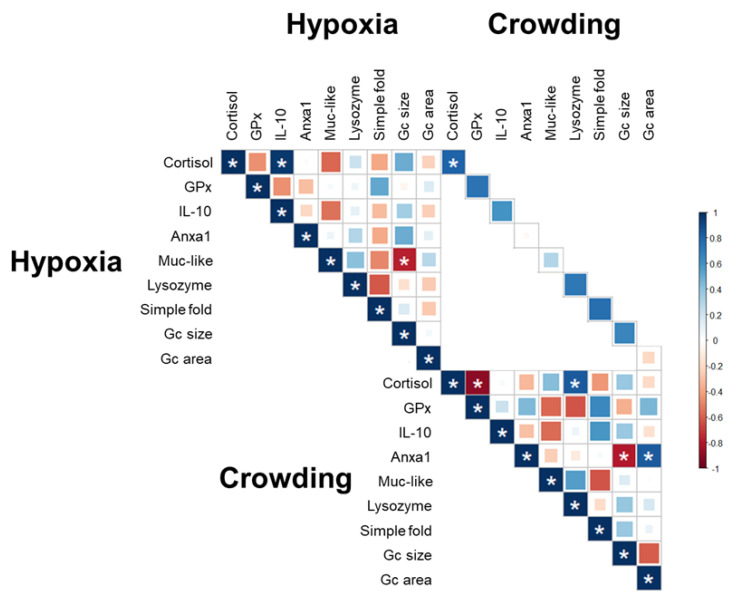
Correlation coefficient between different phenotypic parameters. *: significant values (*p* < 0.05). Degrees of freedom = 5.

Significant correlation of the different parameters (Figure 6) associated with hypoxia stress showed a proportional relationship between cortisol and IL-10 in plasma and an inverse correlation between Muc-like protein in skin mucus and simple fold length in the DI. In the crowding stress group, there was a proportional relationship between plasma cortisol and skin mucus lysozyme and between Anxa1 in plasma and the percentage of simple fold epithelium occupied by goblet cells. Also, in the crowding stress group an inverse correlation was detected between cortisol and GPx activity and Anxa1 and simple fold length. Additionally, there was a significant positive correlation for plasma cortisol between the hypoxia and density stress groups.

## 3. Discussion

In aquaculture husbandry, fish are continually exposed to acute stress conditions such as short-term hypoxia and high crowding densities [1,2]; however, there are limits on the extent to which fish can adapt to these challenges and accommodate the stress response without compromising their well-being. To achieve such adaptation, the mucosal surfaces are important because they are the physical barriers between fish and environment and coordinate the responses against different stressors together with the immune system [3,18]. 

In the present study, we compared two acute stressors (hypoxia and crowding stress) and measured the responses of Atlantic salmon using different biomarkers such as plasma-circulating markers (cortisol, GPx, IL-10, and Anxa1) and mucosal barrier function parameters (Muc-like protein and lysozyme) in the skin mucus, together with histological and morphometric-assessment of DI mucosa. Our study showed some key differences in the measured parameters as effects of these two stressors. 

Cortisol, the primary circulating glucocorticoid hormone, is the most widely used biomarker for stress determination in both mammals and fish. In the current study, cortisol release levels for both stressors were in line with earlier studies in Atlantic salmon, showing the plasma cortisol peak at 1 h after stressful events [19]. In other species, exposure to stressful disturbances resulted in a slightly different plasma cortisol peak. In goldfish (*Carassius auratus*) subjected to hypoxia for two minutes, higher plasma cortisol levels were detected at 30 min and 4 h post-stress exposure [20]. In addition, it has been shown that crowding stress (36 kg m^−3^) in European sea bass (*Dicentrarchus labrax*) also resulted in increased cortisol levels compared to fish kept for 70 days at lower stocking densities (5.5 kg m^−3^) [21].

After the initial increase in plasma cortisol levels, a gradual decrease of cortisol was observed in both models, reaching levels comparable to the initial controls within 24 h post-stress exposure. This response pattern has also been reported in rainbow trout (*Oncorhynchus mykiss*) [22,23,24]. The attenuation of plasma cortisol induced by stress factors can be mediated by a negative feedback loop involved in the regulation of plasma cortisol through the modulation of the glucocorticoid receptor (GR) [22]. Cortisol has been shown to induce the production of reactive oxygen species (ROS) in salmonids through nongenomic pathways [25]. An imbalance in ROS production and the inability of an animal to control excess free radicals can induce oxidative stress [4]. However, fish are known to have developed an antioxidant enzyme system to reduce ROS. Among these enzymes, GPx is a key protein that protects fish from oxidative stress [26]. Our data support this idea, as we detected significantly increased levels of GPx activity at 12 h post-hypoxia stress. These results suggest that fish can prevent or control pathophysiological changes caused by stressful conditions through the enzymatic activity of proteins associated with antioxidant systems. 

Acute stress conditions can also affect the fish mucosa sites through the modulation of components associated with immune responses [27]. We evaluated biomarkers at the phenotypic level associated with both regulatory molecules (IL-10 and Anxa1) and effectors (such as lysozyme and Muc-like proteins) in the innate immune system of Atlantic salmon. In plasma samples, it was noteworthy that with hypoxic stress there was a significant increase in the availability of IL-10 at 1 h post-stress. IL-10, a type II α-helical cytokine, acts as a regulator of processes associated with immunosuppression [28]. Elevated cortisol levels can induce increased IL-10 in mammals [29] and our results are in line with this statement, showing a significant correlation between the plasma levels of cortisol and IL-10 in the hypoxia stress model.

Remarkably, only the crowding stress group displayed significant changes in skin mucus samples. Skin mucus is a barrier that acts as the first external defense, protecting fish from environmental fluctuations and modulating osmoregulation, respiration, nutrition, and hydrodynamic processes. This barrier is formed of glycoproteins, such as mucins, and antibacterial agents, such as antimicrobial polypeptides, immunoglobulin, and lysozyme [30]. The composition of skin mucus is influenced by exogenous factors including stress conditions [31]. In a study with Atlantic salmon [32], it was reported that skin mucus was modulated by stressful situations and our data are in line with this finding, as increased levels of Muc-like proteins were detected at 0, 1, and 24 h post-density stress. These changes in mucin levels may be associated with the higher physical contact between fish at higher densities. The increased physical contact can augment the risk of mechanical damage leading to higher mucus secretion. In addition, we hypothesize that the mucus secretion leads to an increased level of lysozyme, an enzyme with lytic and opsonin activity affecting different types of bacteria [33], at least during the early period of crowding stress (1 h post-stress). However, we did not measure the amount of mucus secreted. The highest detection value for lysozyme was concurrent with a significant increase of Muc-like proteins and plasma cortisol and we also demonstrated a positive correlation with cortisol. This observation is in line with other reports that have presented a relationship between the plasma levels of cortisol and skin mucus lysozyme in salmonids [34,35].

Morphometric changes in the DI suggested a response related to mucus secretion post-acute stress. Previous studies with Atlantic salmon [36] showed that cortisol can modulate intestinal mucosal response; therefore, we suggest a significant decrease in the size of PAS+ goblet cells at 0 and 1 h post-hypoxia may be related to the immediate secretion of mucus triggered by cortisol. This assumption is supported by the negative correlation observed between goblet cell size and Muc-like proteins in the hypoxia model. Goblet cells are mucus-secreting cells that can produce a lubricant for the mucosal surface, which is capable of preventing damage induced by physical or chemical action [37]. In higher vertebrates, goblet cells respond to stress-secreting mucins stored in cytoplasmic granules, leaving small, thin goblet cells that are not easily identifiable [38]. Furthermore, it has been reported that these events can occur around 1 h post-stimulation [39]. Interestingly, we observed an inverse correlation between Anxa1 and the size of goblet cells in crowding stress. In human cell studies [40], annexin II was associated with the secretion of mucin 5ac but its role has not been fully explained. Anxa1 is known to be involved in alteration of intracellular Ca2+ concentration and in induction of apoptosis in salmonids [41], however further studies are needed to understand the role of Anxa1 in post-stress conditions in fish. 

Finally, our results suggest key differences between the two acute stress models and highlight the need for more investigation into the interplay of multistressor conditions and strategies that seek to modulate immunological aspects of mucosal surfaces.

## 4. Materials and Methods

### 4.1. Experimental Design and Sampling

The experiment was carried out at the fish laboratory of the Norwegian University of Life Sciences, Ås, Norway. The experimental fish, coming from the same genetic line and cohort, provided by AquaGen AS (Trondheim, Norway), were fed with a standard commercial diet prior to the experiment. Fish were transferred to experimental tanks and fasted for 48 h preceding the acute stress procedure (hypoxia and crowding stress). The experimental fish had an average body weight of 219 ± 49 g and average length of 26 ± 2 cm (*n* = 78). There were 13 tanks with six fish per tank, each with a 240 L capacity (fish density 6.25 kg m ^−3^), water flow of approximately 8–10 L min^−1^ on average, water temperature of around 15 °C, and continuous 24 h illumination. One tank was assigned as a control group and was not exposed to any type of stressor. The other 12 tanks were divided into two groups and assigned to two different acute stress conditions: treatment A (1 min hypoxia stress) and treatment B (15 min crowding stress). The 1-min hypoxia stress consisted of netting the fish out of the experimental tanks for 1 min and afterwards placing them back into their respective tanks. The 15-min crowding stress consisted of lowering the water level to 20 L and keeping the fish density at 75 kg m^−3^ for 15 min, while maintaining the oxygen level >80% by using an oxygen diffuser. In both groups, fish were sampled at 0 (immediately after stress), 1, 3, 6, 12, and 24 h post-stress exposure, while the control group without stress exposure was sampled only at the 0 h time point.

Per each acute stress condition, six fish per tank (per sampling time) were taken out, sedated (15 mg L^−1^ tricaine methanesulfonate (MS222)), and sampled for blood within 7 min from the moment they were placed into the sedation bath. Skin mucus was gently scraped along the lateral line and collected into s 2 mL cryotube, then immediately snap frozen in liquid nitrogen for further analysis. Approximately 1 mL of blood was withdrawn from the caudal vein using 2 mL heparinized syringes. The blood samples were kept on ice until plasma was separated by centrifugation at 3000× *g* for 7 min. Plasma was aliquoted and placed in sterile Eppendorf tubes to be further stored at −80 °C. Thereafter, the fish were dissected and eviscerated. For histology, samples of distal intestine were placed in 10% neutral buffered formalin for 48 h at room temperature and further processed according to routine histological procedures.

### 4.2. Cortisol

The detection of cortisol in plasma samples (measured in duplicates) was performed using a competitive ELISA Kit (cat no. ab108665, Abcam, Cambridge, UK) following the supplier’s instructions.

### 4.3. Immunological Markers Detection by Indirect ELISA

The total proteins of the skin mucus and plasma samples were quantified using the Pierce BCA Protein Assay Kit (Thermo Fisher Scientific, Rockford, IL, USA), following the manufacturer’s instructions. Thereafter, the detection of immune biomarkers such as Anxa1 (Supplementary materials) and IL-10 in plasma and lysozyme and Muc-like protein in skin mucus was performed by indirect ELISA [42]. Briefly, each plasma sample was diluted in carbonate buffer (60 mM NaHCO3 pH 9.6) and seeded (by duplicate) in a Nunc 96-well plate (Thermo Fisher Scientific, Roskilde, Denmark) (at 50 ng μL^−1^ (100 μL) for overnight incubation at 4 °C. Then, 200 μL of blocking solution (BioRad, Oslo, Norway) was incubated per well for 2 h at 37 °C. Later, the plates were incubated for 90 min at 37 °C with 100 μL of the first antibody (Table 1) and for 60 min at 37 °C with a secondary antibody (100 μL) diluted in a ratio of 1:5000 (goat anti-mouse IgG- Horseradish peroxidase (HRP) or mouse anti-rabbit IgG-HRP). Finally, chromagen substrate 3,3′,5,5′-tetramethylbenzidine single solution (TMB, Thermo Fisher Scientific, Rockford, Illinois, United States) was added (100 μL) and incubated for 20 min at room temperature. The reaction was stopped with 50 μL of 1 N sulfuric acid and read at 450 nm on a SpectraMax microplate reader (Molecular Devices).

### 4.4. Histology and Morphometry

Histological sections were prepared from the formalin-fixed tissues from the DI of fish. The tissues were embedded in paraffin with an orientation that enabled acquisition of longitudinal sections. Sections (2 µm) of paraffin-embedded tissues were mounted on glass slides and processed for staining with haematoxylin and eosin (HE). All tissue sections were also processed for staining with the Periodic acid–Schiff technique. Histological sections were examined under a light microscope (Zeiss Axio Lab.A1, Carl Zeiss, Germany).

Morphometric measurements of the lengths of simple folds in the DI were performed on images of HE-stained sections at 40 times magnification, scanned using a digital scanner, and analyzed using ImageJ software (v1.51r) [45]. The fold height was measured from stratum compactum to the tip of the epithelium lining the fold. Five simple folds were measured from each fish using the segmented line tool. A mean for each individual was calculated based on the five measurements.

For the calculation of the number and average size of goblet cells in the simple fold epithelium, images of PAS-stained sections were analyzed using ImageJ. Five simple folds were selected for HE sections as described above. The freehand selection tool was used to trace the epithelium of the simple fold and a region of interest (ROI) defined. The color deconvolution plugin was used to extract the red (color 2) grayscale image. For the selection of PAS-stained goblet cells, the red image was segmented and threshold settings and particle size settings for detection of PAS staining were then determined and particle count and particle area measurements within the ROIs of each image were performed. A mean goblet cell count and mean goblet cell size in simple folds of the DI for each individual were calculated based on the five measurements.

### 4.5. Statistical Analysis

For calculation of statistical measures and graphical presentation of the results from cortisol, immunological markers, and DI parameters, the software GraphPad v7.03 (San Diego, CA, USA) was used. One-way ANOVA and Dunnett’s multiple comparison test were used to compare each post-stress sampling time with the initial control without stress. In addition, Student’s t-test (two-tailed) was used to determine significant differences between the data for hypoxia and for crowding stress per each post-stress sampling time. Correlation coefficients using the average of each sampling time per group of parameters (per stress model) were performed using the corrplot package in R (available at CRAN: http://cran.r-project.org/package=corrplot). All differences were considered significant when the *p*-value was < 0.05.

## 5. Conclusions

The present study demonstrated that the nature of stress response varies depending on the type of stressor and the method used to induce the stress responses. We observed that the results for cortisol, GPx, and IL-10 in plasma, as well as for goblet cell size in the DI, were more affected by hypoxia stress. On the other hand, crowding stress had a higher effect on Muc-like proteins and lysozyme in the skin mucus. Interestingly, we confirmed a positive correlation between cortisol and IL-10. However, an inverse correlation between cortisol and GPx demonstrated that even a stress event of a short duration triggered enzymes with antioxidative properties as late as 12 h post-hypoxia stress. In addition, the inverse correlation between the goblet cell size and Muc-like proteins during hypoxia, but not during crowding stress, confirmed that there were different coping strategies depending on the type of stressor. Further studies should be undertaken to explore the relationships between different stressors and the stage of the aquaculture production in combination with functional feeds that may modulate the fish’s immune response, thus improving fish welfare.

## Figures and Tables

**Table 1:** List of first antibodies for indirect ELISA.

Marker	Source	Dilution	Reference
IL-10	Mouse	1:400	[43]
Anxa1	Mouse	1:400	Supplementary Figure S1
Muc-like protein	Mouse	1:400	[44]
Lysozyme	Rabbit	1:800	[44]

## Data Availability

The data presented in this study are openly available at this paper. The data that support the findings of this study are available on request from the corresponding authors.

## Supplementary Materials

The following are available online at https://www.mdpi.com/1422-0067/22/3/1028/s1. Validation of antibody against Anxa1. (a) Affinity test by indirect ELISA to establish the proportional relationship between the detection of the antibody produced (at 450 nm) and the concentration of the recombinant Anxa1 (ng μL^−1^). r: Pearson’s correlation coefficient. (b) Three-dimensional modelling by homology. (c) Western blot to determinate the specificity in a plasma sample from Atlantic salmon under crowding stress (pool of four fish).
