# Supplementary figures and images for: Comparison of Circulating Markers and Mucosal Immune Parameters from Skin and Distal Intestine of Atlantic Salmon in Two Models of Acute Stress
Source: Int J Mol Sci. 2021 Jan 21;22(3):1028. doi: 10.3390/ijms22031028 (PMC7864346; doi:10.3390/ijms22031028)

**(a)**

**Indirect ELISA**

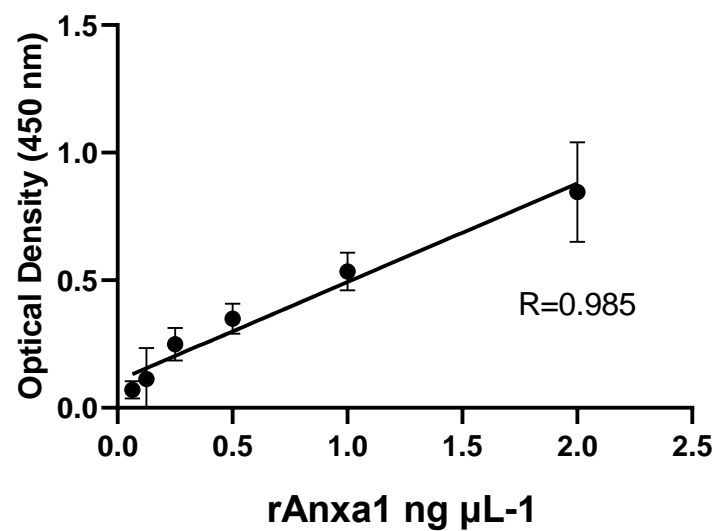

**(b)**

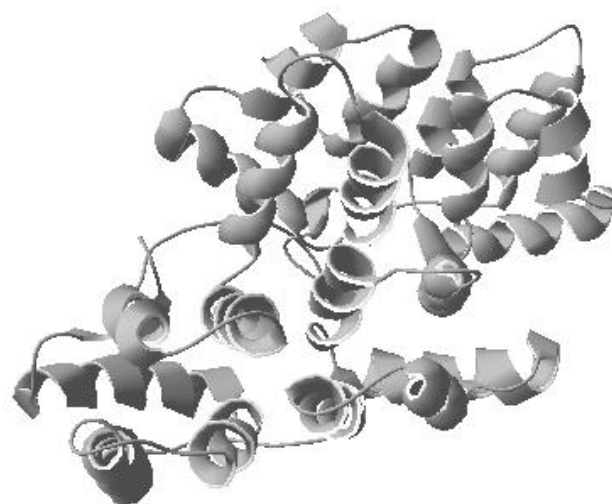

**(c)**

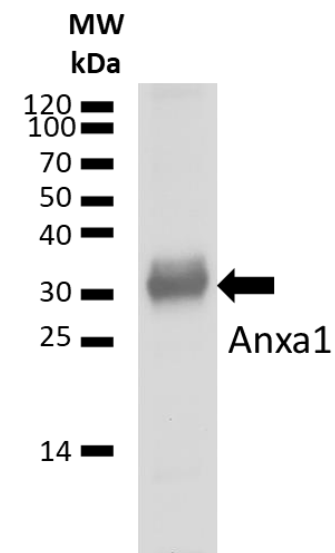

Supplement: Supplementary file 1 [file ijms-22-01028-s001.pdf]
